# Building transformative city-university sustainability partnerships: the Audacious Partnerships Process

**DOI:** 10.1186/s42854-022-00045-5

**Published:** 2023-01-20

**Authors:** Lauren Withycombe Keeler, Fletcher Beaudoin, Abril Cid, Robert Cowley, Samantha Fahy, Amy Lerner, Caroline Moran, Diarmuid Torney

**Affiliations:** 1 School for the Future of Innovation in Society, Arizona State University, Tempe, AZ USA; 2 Portland State University, Portland, Or USA; 3 Universidad Nacional Autónoma de México, MX Mexico City, Mexico; 4 Kings College London, London, UK; 5 Dublin City University, IE Dublin, Ireland

## Abstract

City governments and urban universities are well-positioned to play critical roles in advancing urban sustainability transformations. However, in partnering, cities and universities often focus efforts on discrete sustainability-related projects, neglecting the development of long-term relationships and deep, inter-organizational ties that can allow for collaboration on lasting and transformational change. Yet, at both cities and universities there are often individuals who are deeply interested in developing better partnerships that contribute to the sustainability and livability of their communities. This research develops and tests an evidence-based and facilitated process to guide sustainability researchers and municipal practitioners in the development of transformational City-university partnerships for sustainability. The Audacious Partnerships Process was tested by four City-university partnerships including Arizona State University and the City of Tempe, Dublin City University and the City of Dublin, King’s College London and the City of Westminster and the National Autonomous University of Mexico and Mexico City. The Audacious Partnerships Process as well as results from post-surveys and interviews following implementation are elaborated. We conclude with key lessons for modifying and implementing the process to contribute to transformative partnership development.

**Supplementary Information:**

The online version contains supplementary material available at 10.1186/s42854-022-00045-5.

## Policy and practice recommendations

- Urban sustainability transformations require deep and durable partnerships between cities and universities.
- Before creating sustainability-related collaborative projects, City-university partnerships should first formalize and strengthen the relationship between the organizations.
- The Audacious Partnerships Process can be used by new and existing City-university partnerships to have important conversations that build more transformative partnerships.
- Transformative City-university partnerships must address historic and systemic issues, identify shared values across organizations, and agree to specific and measurable sustainability goals they wish to achieve together.

## Introduction

The sustainability challenges facing our planet continue to accelerate; and, as the world urbanizes, cities can be the source of solutions (Whitehead, 2012; Joss 2015; UN 2017). However, as cities take action, these efforts can fail to contribute to larger system transformation (Ferrer-Ballas et al. 2008; AtKisson 2012; Abson et al. 2017). To move beyond high volumes of short-term, minimally-connected activities (Joss, 2015) and fragmented urban experiments (Evans et al., 2016; Caprotti and Cowley, 2017), and towards more substantial transformations (Evans et al., 2021), it is tempting to look first towards enabling policy frameworks, with City governments playing a critical steering role in partnership with other actors. However, progress towards transformations may be compromised by the fluidity of these contemporary partnership-based arrangements. This article starts with the ‘governance of governance’ (Kooiman and Jentoft, 2009) proposition – suggesting that the design and coordination of collaborative structures is a key success factor in partnership development that encompasses the need to develop trust over time, alongside shared and well-defined values and goals (Ansell and Gash, 2008; Pattberg & Wilderberg, 2016), and capacity to endure in the face of change (Keeler et al. 2018).

City governments and urban universities are well-positioned to play critical roles in advancing urban sustainability transformations (Trencher et al. 2014; Wooltorton et al. 2015; Soini et al. 2018; Purcell et al. 2019). Both types of institutions have significant organizational capacity, plan and take action on long time horizons, and have deep commitments to their communities (Birch et al. 2013; Cuesta-Claros et al 2021). They both face challenges to collaboration as well, such as rigid structures, limited capacity to adapt and, in some cases, tensions from past and current collaborations (Zielke et al. 2021). There is an opportunity to accelerate positive sustainability transformations within urban systems by bringing universities and cities together in ways that intentionally overcomes historic and structural limitations, and which leverage their collective strengths.

City-University partnerships (CUPs) are common, but also tend to be narrowly focused on specific projects in research, development, or workforce training (Caughman et al. 2020). While these can each have sustainability aspects, it is less common to find CUPs that are explicitly organized to center and advance *transformational* sustainability goals, such as completely decarbonizing or ending energy poverty in their city (Trencher et al. 2014; Keeler et al. 2018, 2019). Such goals can be served by joint research projects and workforce training (for example), but the underlying sustainability problems are not solved by those activities because the problems are systemic and they require reconfiguring socio-technical systems through technical interventions, policy changes and behavior change (among others). In short, they require transformation, and that takes time. We argue in this paper that City-university partnerships must be *transformative* in order to effectively contribute to sustainability transformations over long time horizons. Transformative partnerships therefore, are long-term relationships between organizations and the people in them. And to establish those long-term relationships, critical and deep relationship development work needs to be foregrounded.

This paper begins by elaborating the concept of transformative partnerships, then offers a process to structure the development of transformative partnerships between cities and universities, and presents results and reflections from initial implementation of this process in four existing CUPs in four different countries.

### Transformative Partnerships

Transformative CUPs (heretofore transformative partnerships) are partnerships capable of substantially contributing to sustainability transformation in their shared communities. They are partnerships that build intra-organizational “transformative capacity” (Keeler et al. 2019; 2022). Transformative capacity in individuals is determined by sustainability competence (Wiek et al. 2011; Meharg 2020); confidence in executing sustainability activities, often gained through experimentation and double-loop learning (Von Wirth et al. 2019; Smith 2012); commitment over time to seeing sustainability transformation through; and the power to turn ideas into action (Avelino 2017; Avelino and Rotmans 2011). Transformative partnerships build the transformative capacity of their organizations by expanding the capabilities of individuals in the partnership by building sustainability competence in the organizations, developing the confidence of individuals and groups to contribute to long-term sustainability transformations, creating shared commitment to achieving sustainability goals, and bringing power to bare in the solving of sustainability problems (Keeler et al. 2019, 2022; Meharg 2020). The challenge is how to design, implement, and maintain partnership structures that build transformative capacity, rather than temporary coalitions determined by the needs of individual projects.

A seminal example of a transformative CUP is described by Allen and colleagues (2017). This work details the goals and design process for developing a CUP that centered transformative progress on climate action in the City of Portland. This partnership process began by focusing on the systems that would allow for a long-term, mutual and impactful relationship. These systems included: processes for allocating resources to projects, agreements for how to collaboratively seek funding, monitoring and evaluation and clear role delineation. Allen et al. (2017) draw a direct connection between the forethought and energy put into the partnership and the quality of the projects that resulted (many of which produced significant sustainability outcomes for the City of Portland). One of the projects highlighted in the paper focused on passing a deconstruction ordinance in the City (a policy requiring buildings built before a certain date to be deconstructed  rather than demolished). This transformational outcome came as the result of a strong partnership foundation, which included: (i) A process for quickly accessing new money to catalyze and evolve the project into new phases (to respond to new policy questions and needs). This allowed funding to be pursued in response to shared, transformational sustainability goals, rather than in response to funding availability and the values of funding organizations. And, (ii) the networks and capacity to bring in new actors into the work to fill unforeseen knowledge gaps and building the sustainability competence of the partnership. After multiple adaptations to the project (and numerous deliverables) the policy was passed^1^; it has been in place for 6 years and resulted in over 550 house deconstructions and 7.6 metric tons of saved carbon (taking over 900 cars off the road). The example shows the link between a focus on transformative partnership development and transformational sustainability outcomes. Achieving transformative sustainability outcomes through CUPs is complex and contingent on questions of governance. To improve CUPs, the processes used to design and implement partnerships need to center transformations, and build and maintain the structures needed to support progress towards those transformations.

This paper integrates research conducted from 2017–2021 in the CapaCities network, an international network of CUPs, on building capacity in cities, universities, and their partnerships to contribute to urban sustainability transformations. The CapaCities network includes: Arizona State University (ASU) and the City of Tempe, USA, Dublin City University (DCU) and the City of Dublin, IE, Karlsruhe Institute of Technology (KIT) and the City of Karlsruhe, DE; Kings College London (KCL) and the City of Westminster; Leuphana University (LUL) and the City of Lueneburg, DE, the National Autonomous University of Mexico (UNAM) and Mexico City, MX; and Portland State University (PSU) and the City of Portland, USA. The network has conducted case studies and cross-case comparisons on the functioning of CUPs and developed and tested new methods and approaches for building transformative capacity. Results include critical characteristics for effective City-university partnerships, (Keeler et al. 2018) a theory of actor-centric transformative capacity which, when developed in City staff, advances urban sustainability transformations (Keeler et al. 2019) and a framework and method to formatively evaluate and adapt transformative City-university partnerships (Caughman et al. 2020). This research integrates these results into a framework called the Audacious Partnerships Process. This paper describes the development of the Audacious Partnerships Process, its implementation in four CapaCities Partnerships: ASU-Tempe, DCU-Dublin, KCL-Westminster, and UNAM-Mexico City, and results of a shared survey instrument and focus groups implemented across the cases. The discussion and conclusion offer reflections on limitations and how the framework can be used to help other CUPs move toward sustainability transformations.

### Transformative sustainability partnership development

City-university partnerships (CUPs) have the potential to advance sustainability transformations (Giunta and Thomas, 2013; Kreuter et al. 2000), but the literature does not offer many examples of long-term CUPs that have achieved this goal. The Audacious Partnerships Process was developed based on the research and experience of the CapaCities network, particularly transformative capacity building, and existing literature on partnership development for sustainability. The Audacious Partnership Process includes three stages: Building Organizational Teams; Facilitating Partnership Development; Follow up and Implementation. The stages are designed to surface and facilitate conversations and (subsequent actions) to address historical and structural limitations at the organization and partnership levels so that partnerships can support progress towards sustainability transformations. The theory of change guiding this work is simply that transformative partnerships require a focus on (and foregrounding of) relationship development and maintenance and an orientation toward transformational and shared sustainability goals. Attention to these specific areas builds the transformative capacity of CUPs. The Audacious Partnerships Process guides participants in designing the structure and function of the City-university partnership around co-defined, shared values and transformational goals. These values and goals are upheld by supporting structures to maintain the healthy functioning of the partnership and which are identified during the process as most critical to the organizations and individuals involved in the partnership. Values, goals and structures are then reinforced through co-designed and impactful actions which can be owned by individuals and teams through their roles and responsibilities and which demonstrate the translation of relationship development into practical sustainability-related action, while building transformative capacity (Table 1). The process was designed to address these factors through a series of facilitated, partnership development activities.

**Table 1:** Enabling factors that build the transformative capacity of City university partnerships

ENABLING FACTORS	DESCRIPTION
*ADDRESS HISTORY AND PAST CONFLICT TO CREATE A FOUNDATION OF TRUST AND RESPECT*	Quite often new City-university collaborations are forged without a clear understanding of the organizational history and challenges that can can be the source of trust and mistrust. This may result from a desire to focus on the future (Kalayjian and Paloutzian 2009), a lack of understanding of current and former collaborative relationships between the organizations (Zielke et al. 2021; Keeler et al. 2018), or a sense that because individuals involved in the current partnership did not take part in activities (that engendered mistrust) that it is not their responsibility. A transformative partnership is a long-term effort and therefore needs to be built on strong relationships and a foundation of trust (Allen et al. 2017). Part of building that trust requires accounting for and addressing past wrongs. If this factor is not addressed up front it corrodes the potential impact of the partnership
*EXPAND THE PARTNERSHIP TO ENCOMPASS TRANSFORMATIONAL GOALS THAT CAN INFORM DISCRETE* *PROJECTS*	City-university partnerships most often form around discrete actions and programs with specific timeline and deliverables. This approach can get a collaboration moving, but can miss the opportunity to see and organize around the larger transformation. When facing sustainability crises, scholars argue that we must take a “systemic and integrated” approach to the problem or we will fail to address the pernicious root causes (Yarime 2012)
*INVEST IN THE RELATIONSHIPS THAT UNDERPIN THE PARTNERSHIP*	In a City-university partnership there is a limited amount of energy that collaborators provide. Quite often the energy will be heavily weighted towards the specific project – which constrains the amount of time needed for relationship development. Achieving transformative outcomes together requires shared commitment engendered through relationship development at multiple levels of each organization. A transformative partnership requires that time be spent building relationships and understanding between people across organizations in order to motivate long-term engagement in the partnership and the sustainability transformations sought (Allen et al. 2017; Caughman et al. 2020)
*ESTABLISH AND MAINTAIN THE NORMS AND STRUCTURES THAT SUPPORT TRANSFORMATION*	Organizational norms and processes are needed to facilitate long-term, cross-organizational collaborative work and these often do not exist (or are not attended to) in City-university partnerships. This can be specific to engaging in the partnership or specific to the capabilities of the organization. For example, universities may not incentivize engaging in partnership activities because they do not translate directly to traditional metrics of academic success, such as publications. Cities can experience high turnover, scarce resources, or lack of capacity that limit their ability to support engagement in partnership activities over the long term. Overcoming this limitation requires organizational transformations that redefine roles, structures and norms to address the complex sustainability challenge (Keeler et al. 2018)
*DESIGN ACTIONS THAT ARE DEEPLY MOTIVATING*	Research on City-university partnerships has shown that collaborations are more durable and effective when actors on both sides of the partnership have high levels of motivation to engage (Keeler et al. 2018; Caughman et al. 2020). Motivation comes from engaging in collaborative activities that are mutually beneficial and perceived to have an impact. When motivation is lacking, the collaborative work demands too much of a single collaborator (the City or the university stakeholders); this limits the capacity for co-creation and can fracture the relationship

### The Audacious Partnerships Process

The Audacious Partnerships Process was originally designed as a series of in-person engagements culminating with a board game played by the City-university partnership teams. The process and game were developed in the Fall of 2019 and tested at Dublin City University with ASU, DCU, KCL, KIT, UNAM, PSU and City of Dublin partners. The boardgame was finalized in March 2020 but was never used in-person because of COVID – 19 restrictions. The framework was adapted for application online, utilizing Zoom for video conferencing and Mural for engagement (an on-line platform for visualizing and collaboratively manipulating information and objects). The three stages of the Audacious Partnerships Process, summarized in this section, work together to layer the conversations and activities to deepen relationships and generate momentum over time. See supplementary material for a copy of the facilitation guide which elaborates the stages and key activities in the Audacious Partnerships Process.

#### Stage 1: Building organizational teams

An individual or small team begins by identifying university and City stakeholders to engage in the process. This phase introduces participants to the Audacious Partnerships Process, its motivations, and its aims. Teams can include university staff, faculty, students, and City staff, administrators, and leaders. By the end of this phase, a small but committed coordinating team of 1–2 people from each organization should be formed and a larger group to guide the partnership should be identified, and buy-in for the process should be secured from all participants. Separate meetings are held with each organizational team: City and university. In these meetings, the teams will identify the core values they want to guide the partnership and the transformational goals they are prepared to work toward. These are integrated into a team profile that is shared with the other organization as way of introduction prior to meeting (see supplementary material). These meetings also help the organizational teams to get to know one another, they help turn a group of individuals into a team. Teams are also able to familiarize themselves with the kinds of conversations that will take place in the partnership engagement.

#### Stage 2: Facilitating partnership development

Stage 2 is the main series of facilitated activities in the Audacious Partnerships Process. Teams from the City and the university come together for a workshop. Both teams bring the values and goals they established in stage 2 and are guided through a facilitated process to share and explain those values and goals. The teams explore places of synergies and conflicts. The teams are then encouraged to identify shared values to guide the partnership, and agree upon transformational sustainability goals to work toward. A second, facilitated activity guides the teams in identifying structures that will help maintain the relationship between the City and the university, summarized in Table 2. Together participants answer guiding questions about each structure and prioritize the structures that will be most important for long-term partnership development and maintenance.

**Table 2:** Supporting Structures for Transformative Partnerships, adapted from Keeler et al. 2018 and Caughman et al. 2020

Structure	Definition	Guiding Question
Motivation	The drive from stakeholders to engage deeply in the partnership and sustain that engagement over time	What needs to be in place to motivate your continued engagement in this partnership?
Reflexivity	The feedback mechanism that ensures partnership goals and structures are maintained and can evolve	How would each partner like to receive feedback and what feedback would help the partnership to achieve its unique goals?
Resources	The financial, political, and human capital needed to support and grow the partnership	What resources does your partnership need to achieve your unique partnership goals?
Process	The formal and informal mechanisms and agreements that support the effectiveness of the partnership	What processes need to be in place for you to achieve your unique partnership goals?
Participation	The clear engagement of people at different levels within each organization and pathways for new individuals to become involved	Who needs to be involved in the partnership and how can multiple pathways to participation be established and maintained?
Understanding	Shared understanding of cultures, demands, values, and histories between and among partner organizations	What do you need your partners to understand about you and your organization to achieve your partnership goals?

#### Stage 3: Follow up and implementation

This last stage focuses on activating the work from Stage 2. Teams come together in a reflection session to process the results of the workshop, add additional insights, and discuss how to implement key activities to move the partnership forward. This stage produces a clear set of actions that the teams believe are most critical to the formation of a transformative partnership. These actions below are offered as possible next steps, though partnerships are also encouraged to identify their own actions:

- Identifying long-term goals
- Creating a work plan for the partnership
- Developing criteria for projects
- Publishing results from the partnership
- Hosting trainings on transformative partnerships
- Building a core partnership team
- Documenting characteristics of other partnerships
- Building pathways to engage more collaborators
- Hosting information sessions about the partnership
- Developing pathways for students to engage
- Developing talking points for the partnership
- Presenting at a conference or event
- Mapping critical stakeholders
- Developing a partnership charter
- Launching a project together
- Evaluating past projects
- Identifying internal resources for the partnership
- Applying for external funding
- Creating a new position to support the partnership
- Leveraging existing positions to support the partnership

The three stages of the Audacious Partnerships Process are designed to build transformative capacity among the participating partners. The design considerations and objectives are summarized in Table 3.

**Table 3:** How the three stages of the Audacious Partnerships Process enable transformative partnership development

Enabling factors for transformative partnerships	General	Stage 1: building the team	Stage 2: facilitating partnership development	Stage 3: follow-up and implementation
*ADDRESS HISTORY AND PAST CONFLICT TO CREATE A FOUNDATION OF TRUST AND RESPECT*	Centering the need to have important conversations about the past, present and future throughout the process	Growing an initial team that has experience working with the other organization and enough trust, understanding and interest to have honest conversations about the past and what comes next	Specific questions and prompts that learn into the hard conversations about the partner’s past and future	Unpacking the challenges and tensions that arose during the process so those lessons can be woven into implementation
*EXPAND THE PARTNERSHIP TO ENCOMPASS TRANSFORMATIONAL GOALS THAT CAN INFORM DISCRETE PROJECTS*	Starting with conversations about the broader relationship before digging into specific projects	The team should have the capacity to think creatively and with optimism about what is possible	The process begins with (and then centers throughout) the future vision that is possible between the two organizations	The actions taken after are filtered through the larger vision and set of possibilities for the partnership
*INVEST IN THE RELATIONSHIPS THAT UNDERPIN THE PARTNERSHIP*	Creating informal space that allows for learning about the individuals and their interests, but also enough formality so action can be taken	The team is built based on people who have project experience, but also the capacity to think and work at the partnership level	The process begins with a discussion of sustainability goals and values so a foundation for the partnership can be set before exploring projects	Initial implementation actions center around activities that feed the development of critical elements of the partnership
*ESTABLISH AND MAINTAIN THE NORMS AND STRUCTURES THAT SUPPORT TRANSFORMATION*	Ensuring that conversations are unpacking and addressing historic and structural limitations that prevent transformative outcomes	The team must be aware of (but not overwhelmed by) the systemic issues that currently limit the partnership	The process centers the key structures that need to be in place to help overcome some of the structural and historic limitations	Implementation efforts center activities that can develop the structures and relationships that grow the partnership’s capacity to overcome limitations to transformation
*DESIGN ACTIONS THAT ARE DEEPLY MOTIVATING*	Deeply exploring core values, interests and the resources of the organizations and individuals involved so actions are implementable and sustainable	The teams should see clear motivation for themselves and their organization in the work to build a transformative partnership	The process helps unpack and clarify true areas of alignment and interest across organizations and helps uncover projects and activities in those areas	Implementation is about activating the areas that have the strongest motivation and ability for a team to catalyze, adapt and sustain

## Research approach

To test the viability and initial effectiveness of the Audacious Partnerships Process, the process was implemented in parallel at four CUPs. The goal was to understand how the process functioned in practice and in different contexts. The CUPs differed in the breath, depth, and centralization of the existing relationship (and the extent to which discussions over values had already taken place). While a standardized approach was implemented to allow for comparative analysis, the process was adapted by each partner to reflect their context and needs. Guidelines for implementation included:

- Utilize all four stages of conversations that intentionally build on each other towards the most important conversations and a motivated team.
- Develop a core team from the City and university to initiate the work, and bring in between 5–7 more individuals from the partnership to participate in the joint conversation during Stage 3.
- Use a facilitator to support the City and university with resources and consultation for each stage.
- Implement a post survey to evaluate the impact of the process.

University partners were encouraged to adhere as closely to the spirit of the process as possible, but to document adaptations that were used to make the process functional for their case. In the case of UNAM, adaptation necessarily included translation to Spanish but also included adaptation of concepts and stages for the Mexican context (see Temper et al. 2019).

To understand how the process impacted the City-university partnerships, a post-survey was administered to City and university participants in the Audacious Partnerships Process after the conclusion of Stage 3. The survey included 6 statements about the process and asked participants to evaluate those statements on a 5-point Likert-type scale (questions and Likert- scale are summarized in Table 4). Participants were also asked three open-ended questions about their experience with the process (Table 4).

**Table 4:** Post-survey questions administered to all participants after Stage 3 of the Audacious Partnerships Process

*Prompt or Question*	*Response Format*
• *Engaging in the Audacious Partnerships framework helped our group have important conversations*	• Agree • Agree somewhat • Neither agree nor disagree • Disagree somewhat • Disagree
• *The Audacious Partnerships framework made me think about aspects of our City-university partnership that I hadn’t thought about before*	
• *I learned something new about our partners during play*	
• *The audacious partnership framework helped me see ways our partnership could be improved*	
• *The Audacious Partnerships framework helped make progress toward designing a more transformative City-university partnership*	
• *I am more motivated to develop a transformative City-university partnership than I was before engaging in the Audacious Partnership sessions*	
• *What were some positive or helpful moments during the City-university session?*	Open ended
• *What were some challenging or difficult moments?*	
• *Any additional feedback you’d like to provide?*	

To substantiate the post-survey, individual case interviews were conducted by a Portland State University researcher after reviewing the results of the survey. University participants were asked to elaborate on the responses given in the survey in order to elucidate the substance of the conversations throughout the process. Interview questions included:

1. Describe how the Audacious Partnerships Process was implemented in your case.
2. How closely were you able to adhere to the process?
3. Where and how did you adapt?
4. What if any important conversations were had?
5. What were important takeaways from the process?
6. What are next steps for the City-university partnership?

Finally, a focus group was held with all participating university researchers to discuss the results of the case implementation and identify shared insights and divergent experiences.

## Case descriptions

### Arizona State University and Tempe

Arizona State University’s largest campus (~ 55,000 students) is in Tempe, Arizona (pop. 187454). The City and the university have a long history of collaborating on sustainability research, engagement and educational activities and significant connections between the two institutions (alumni that work at the City, City stakeholders that City on university committees, etc.). Most of the sustainability partnerships between the City and the university are managed at a project level, meaning that there are multiple touch points between City and university stakeholders working on a variety of individual activities and deliverables. The university is also a significant landholder in the City of Tempe and their influence can be a source of tension and can sometimes conflict with ostensibly shared sustainability goals. This tension came up throughout the process. Only a few stakeholders are thinking about the health and functioning of the overarching partnership. These stakeholders (an assistant professor and the sustainability director at the City of Tempe) were the core actors that instigated conversations and activities around the Audacious Partnerships Process.

### King’s College London and Westminster

King’s College London is in London, UK (serving approximately 27,000 students). Its campus is spread across three central local authority areas, including the City of Westminster (with a population of c.234,100). In April 2019, the university and Westminster City Council (WCC) signed a high-level ‘Statement of Intent’, to take a longer view of mutually beneficial collaboration. Before then, a range of relevant research, education and engagement projects had taken place. However, these were fragmented across different parts of the university and City, without support from an active strategy to ensure the health and functioning of the overarching partnership. For this case, there was one central stakeholder (a lecturer at Kings College) who catalyzed the use of the Audacious Partnership Process.

### Dublin City University and Dublin

Dublin City University is located in Dublin, Ireland. The population of Dublin is 1.43 million people and the University serves to over 18,000 students. The City and the university have a history of collaboration, but limited examples of partnerships that have been sustained over time. Dublin is also home to other major universities who also engage with the Dublin City Council (the City). The core stakeholders that drove the project were a sustainability staff person from the University, a professor and a PhD student.

### UNAM and Mexico City

UNAM is the largest University in Mexico City, MX. The City has a population of approximately 9 million, a metro area of approximately 22 million, and the University serves over 360,000 students in total, with over 110,000 in the Mexico City campus: A mega-University within a mega-City. The locus of this collaboration was between the National Laboratory of Sustainable Science within the Institute of Ecology (LANCIS-IE) in UNAM and the Resilience Department of the Secretariat of Risk Management and Civil Protection in the Mexico City government. Overall, the University is well-respected within the City, region and country. There is a long history of collaboration on projects between the City government and the University on a wide range of topics; these collaborations are messy, wide-reaching and not managed centrally. The key actors for implementing the process were an Assistant Professor at UNAM, a PhD student, and a City staff person in the Resilience Department (who also was a graduate student at UNAM).

## Results

### Survey results

**Fig. 1:**
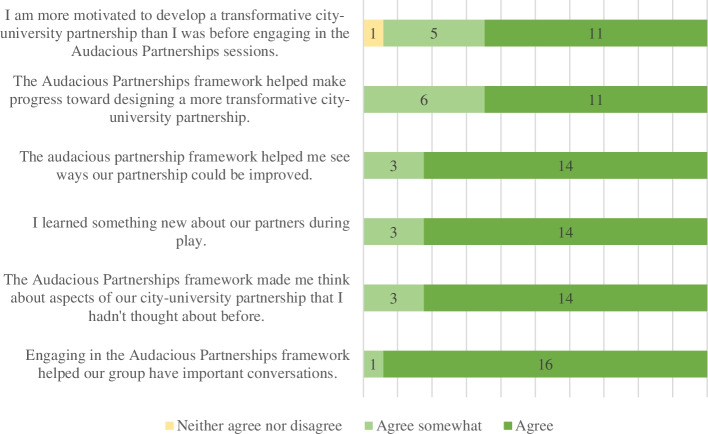
Distribution of Likert-type responses to survey statements which demonstrate that substantial agreement with survey statements

**Fig. 2:**
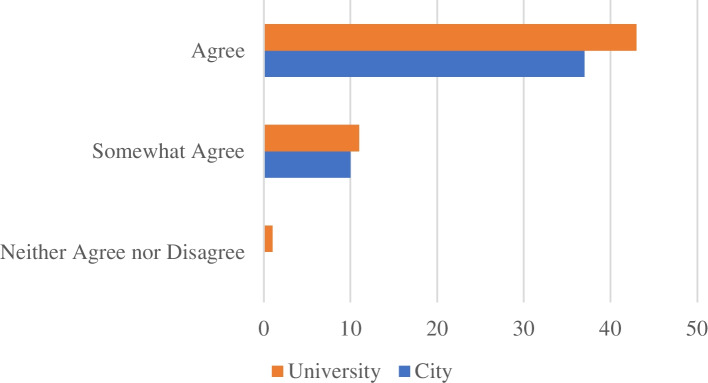
Distribution of Likert-type responses to statements by University and City participants

Survey results were collected from three of the four participating City-university partnerships. UNAM determined that it was not appropriate for their case study to implement the post-survey. Instead, a more in-depth debrief was conducted with UNAM researchers and results from that debrief appear in the cross-case comparison and discussion. From the three-participating City-university partnerships, Likert-type responses to statements were overwhelmingly positive (Fig. 1). Across the three City-university partnerships 17 survey responses were collected from 8 City participants and 9 university participants (Fig. 2).

Responses to the survey indicate there was general agreement between university and City partners that the process was useful for facilitating important conversations, learning new things about the partners and identifying ways to improve the partnership. However, results about creating a more transformative partnership were more guarded. In particular, only half of City partners agreed with the statement that the process left them more motivated to develop a transformative City-university partnership. The one respondent who selected “neither agree nor disagree” for this statement commented that they found themselves preoccupied with reading the board game material and would have liked the material in advance so they could better participate. This affected their ability to engage and therefore their motivation to continue engaging in such a process. Another respondent noted that while they were told of the time commitment for engaging in the facilitated portion of the Audacious Partnerships Process, they were not aware that there would be an ongoing commitment and could not make such a commitment without permission from their department.

### Cross case analysis

This section synthesizes results from the surveys and focus groups with the university teams during and after engagement in the Audacious Partnerships Process. This section aims to detail how to use the Audacious Partnerships Process to build more transformational partnerships, what challenges can arise in so doing, and what limitations were experienced during implementation.

#### The audacious partnerships process requires identifying key stakeholders who are committed to a vision of long-term and transformative partnership development

While the process can help build commitment to the partnership, it also relies on existing commitments to sustainability and collaboration. Transformative capacity requires commitment (see Table 1) and this commitment can be engendered by developing norms and reward systems that connect the efforts of individuals to larger goals. However, implementing the Audacious Partnerships Process made clear that it is far easier to begin partnership development with people who are ready and motivated to be part of the process. ASU, KCL, and DCU worked with one or more City partners identifying who was best-suited and most-needed from each organization for Audacious Partnership process. At DCU, the team took the time to ensure that the process and results translated into the Irish cultural and professional context. They were aware that the process could push City stakeholders out of their comfort zone, which led them to recruit of partners at the City with whom they had existing relationships and trust. This proved to be a successful strategy. ASU also relied on existing relationships, but with the added focus of identifying partners who were experienced with collaboration. For KCL, it was more important to identify an initial core of stakeholders that were interested in engaging. In contrast, at UNAM, the City partners were completely new from past projects due to a change in the government party at the time, which set a completely different agenda. The Resilience Office (which was the University’s core partner) was transformed and moved during a governmental change that happened in the middle of the process – making it unclear who would be the core contacts. The process served the purpose of identifying new allies for a transformative partnership within the new government.

#### Understanding the partnership history and ongoing collaborations between the organizations sets an important foundation for future success

Because each City and university have some history of interaction, it was important to understand how the organizations collaborated and functioned in the past. Sustainability transformations are long-term endeavors and transformative partnerships are meant to help shepherd decadal-scale change. In reflecting on the Audacious Partnerships Process, participants emphasized that the people involved need to trust one another, and part of building that trust is accounting for past wrongs, as well as, elevating fruitful examples to guide planning for the future. ASU, KCL, and DCU all began by identifying ways in which the City and the university were already collaborating. For ASU, high level takeaways included: a) no clear point person at the university attending to the health and functioning of the partnership; b) the core contact for the City of Tempe is managing multiple relationships and projects with the university and is stretched thin. These are issues that were not being addressed because collaboration had always been project-based. The Audacious Partnerships Process created space to reflect on aspects of past collaborations that did not work and set terms for future collaborations to succeed. At KCL, the focus was on identifying who to talk to at the City and University about this partnership. Since the current relationship was decentralized, this required significant time and energy to find the people and also to build new relationships. DCU focused on understanding the City-university partnership landscape and the motivations and interests of actors within the City and University. The Dublin Metropolitan Climate Action Regional Office, which coordinates climate action within the four Dublin local authorities, was forming just as these conversations began; this Office was chosen as a core partner because of their interest in partnering with DCU and the alignment between their opportunities for partnership and DCU expertise. At UNAM, the partnership between the City and University spans many departments and has a deep history. The group decided to focus on urban sustainability and resilience and use the Audacious Partnerships Process to forward that collaboration. Creating transformative partnerships requires recognizing the good and bad in past collaborations and co-defining ways forward that work for both sides. The Audacious Partnerships Process provided space to begin those conversations, but more time needs to be spent resolving those issues, it cannot all be done during three short meetings.

#### The case for a more intentional city-university partnership is not self-evident and needs to be argued for and reiterated throughout the process

All four university partners had to make the case for building a partnership in an intentional manner at the outset. Transformation requires power – the individuals and groups involved in transformation need to be able to turn their ideas into impactful action. The need for action though can lead to a project-oriented focus. In convening participants in the process, university leads were challenged to justify a focus on partnerships, particularly as City and university participants alike have specific output metrics against which their performance is judged. Creating discursive space to understand underlying values was not something that all participants had particularly considered, and the value of a values-forward approach was not immediately evident. At UNAM, the stakeholders decided to focus the conversations on stakeholders from LANCIS-IE and from the Resilience Office of the City, building on existing relationships and creating the connections necessary to support the ongoing resilience efforts. At DCU, the process of identifying stakeholders at the university and City led to the realization that there was no existing forum for conversations about partnership values and goals. DCU believed it would be challenging to build a process that centered partnership values and goals, so they chose to engage stakeholders who already had connections and trust with the core team. At ASU, making the case for the partnership meant expanding the circle of people at the City and University having conversations about the partnership, its history, and how it could better serve sustainability transformation in their shared communities. In each case, the argument needed to be made that concrete work on sustainability-related projects should happen within an overarching partnership structure so that project outputs can be linked over time to contribute to larger system transformation.

##### The audacious partnerships process held space to work through important, underattended issues

All groups felt that the process facilitated important conversations that would not have otherwise occurred. Discussing shared values and how those values ought to inform how the cities and universities worked together was especially valuable. Building commitment to transformation is a value-laden process, drawing on the missions of organizations and the convictions of individuals. Transformative partnerships need to tap into the values of participants and bring them to the forefront. UNAM expressed openness and hope about the process and opportunity for an expanded partnership and many of the stakeholders immediately saw how these conversations could build capacity for more effective work. At KCL, both conversations with the University and City were described as “productive” and led to a playful curiosity on either side to see if similar values had emerged in the parallel conversation. At ASU, the conversation with the City stakeholders revealed more challenges with the partnership. The City had been dealing with the university as a large landholder, and researchers were unaware of ongoing disputes over particular developments and the university’s advocacy for transportation accommodations that ran counter to the City’s sustainability goals. This tension was affecting a deeper CUP and none of the university participants was aware that a problem existed prior to the meeting. However, the conversation demonstrated that both sides had shared values and were eager to address the issues that arose during the process.

##### The opportunity to unpack organizational structure may be key to overcoming challenges

Organizational design can impede transformation. The design of universities and cities are different but each have limitations that need to be understood and confronted in order to build transformative capacity. The Audacious Partnerships Process revealed these organizational structures and the impediments they reproduce, and to a minor extent, partners began the process of determining how a partnership might overcome structural limitations. At ASU a series of important conversations emerged. First, the university side was made aware of some of the partnership challenges that were more abundantly clear to the City of Tempe stakeholders, e.g. that the university contradicts itself when it engages with the City by advancing small and medium-sized sustainability-related projects on one side, advocating for massive investment in unsustainable development on the other. Second, the education and training opportunity of the partnership was valued highly by the City of Tempe – above the benefit provided by students and researchers, and this could be better leveraged to achieve sustainability goals. At UNAM the teams had strong alignment in their goals and values. The two parties identified the need for partnership coordinators, which could strengthen the longevity of the relationship. Developing a collaborative portfolio of projects was a key action that could allow the partnership to be both reactive and proactive to opportunities. Both City and university partners felt an obligation to and enthusiasm for working with students and on student projects. If effectively shepherded in the Audacious Partnerships Process, student projects could help advance sustainability transformations by providing for safe experimentation, which builds confidence in new and innovative solutions.

##### The process increased confidence among city university partners in collaborating with one another in the future

Transformative capacity requires confidence in people and pathways and commitment to sustained action over time. Personnel changes can undermine transformative partnerships by erasing the locus of action and eroding confidence. The process facilitated conversations about how the people present could work together, and what was needed to ensure new people could join the partnership and sustain the commitment to transformation. At KCL, partners proposed building a coordination role into the Kings College business plan so that someone could be responsible for thinking about and maintaining a partnership between the City and university. This was a significant evolution from initial conversations which necessitated justifying a discussion of transformative partnerships. Participants cited the Audacious Partnerships Process as a model that KCL could use to engage with other London municipalities. After the process at UNAM, however, stakeholders (the PhD student and Assistant Professor) stepped away from the partnership because of new roles. However, the results from the game were summarized and shared with stakeholders in the University and the City and during the process the work of LANCIS-IE was elevated within hierarchy of the City. The core City stakeholder remains and is restarting conversations with LANCIS-IE. As ASU began implementation, the university stakeholder brought on a student to execute an analysis of what has been working, what has been challenging and where there are future opportunities for the partnership. Since engaging in the process a few stakeholders on the City and university sides are no longer at the institutions, but enough remain to continue to make process and build on the initial results. Ideally, a transformative partnership would gird against this kind of disruption in key partnership personnel and keep the collaboration trained on the long-term sustainability goals. While the process established a willingness to engage in further collaboration on a transformative partnership agenda, the vision was not fully realized in time for these personnel departures.

### Analysis of the Audacious Partnerships Process and factors that enable transformative partnership development

The core purpose of the Audacious for Partnerships Process is to center factors that enable transformative capacity building within City-university partnerships through relationship development and a focus on long-term transformation. These cases indicate that the process can help initiate transformative partnership development. The section below outlines each of the enabling factors and discusses how these factors played out in the implementation of the Audacious Partnerships Process.

- *Address history and past conflict to create a foundation of trust and respect:* All of the cases had history and context to address and some had acute challenges from that history. Foregrounding conversations about history and context (and conflict, for some cases) ensured that the conversations were grounded in key issues that must be addressed to center transformations. The process helped stakeholders develop a mutual understanding of the perspectives and constraints facing each organization. These initial conversations must be carried through to actions which demonstrate that historical issues are being addressed, not just talked about.
- *Expand the partnerships to encompass transformational goals that can inform discrete projects:* The process began by centering the larger opportunity (goals, values and structures) of the partnership. Centering the vision and potential for the partnership, ensured the projects and actions were grounded by an inspiring and uniting vision. This vision needs to be preserved by the partnership and returned to and revised as the partnership is maintained. These should be living goals that actively inform actions and collaborative projects and they should be updated as the partnership evolves. *Invest in the relationships that underpin the partnership:* Relationship-building was key to each of the cases. This was a constant theme that ran throughout and a core outcome of the entire process. Since relationship-building a central goal, it changed the engagement process – placing a higher value on clear communication and mutual understanding. These factors all became foundational for the stakeholders to grow closer and build trust. Turnover in personnel can stall a partnership. A transformative partnership should be capable of withstanding turnover while keeping the partnership focus trained on transformational goals. However, we saw turnover during transformative partnership development present a significant hurdle to carrying through on longer term actions to support the partnership.
- *Establish and maintain the norms and structures that support transformation:* Transformations require significant participation, adaptation and long-term support and resources. The different cases held to these pillars by balancing structure with adaptation – ensuring there was enough stability to provide direction, but also flexibility so that new learning and context could shift strategy. Most cases also implemented the process with shared leadership, where multiple participants played leadership roles. This type of leadership allows for a greater diversity of thought and creates pathways for broader engagement in the project – two factors that are critical to advancing complex change. Having multiple leaders on the project also creates built-in partnership resilience when one of the team members leaves or is low capacity (ensuring the work can continue in the face of disruptions).
- *Design actions that are deeply motivating:* A core intention throughout the process was to center the needs and interests of the stakeholders involved in the partnership. This required a strong focus on the development of trust and relationships so that people were comfortable sharing honest comments and ideas. Because of this, many of the cases led to actions that were implemented (because of the high motivation behind the proposed actions).

## Discussion

Both City governments and urban universities have significant impacts on the lives of their shared communities. They are also both values-driven organizations. In implementing the Audacious Partnerships Process we found that there were shared values between cities and universities and that a values-forward conversation could help orient conversations about collaboration toward a higher, longer-term purpose. However, the way those values are enacted through professional responsibilities is different. The structures that propel cities and universities forward sometimes throw them towards collaboration, but sometimes they place them in conflict. That tension came out in discussions of the history of interactions in our CUPs. The Audacious Partnerships Process held the space that surfaced those conflicts but good facilitation was required to work through them, and much was left unresolved. In addition, the participants were selected because of their interest in sustainability and willingness to collaborate, but there are many other actors at either institution-type that do work that intersects with the partnership and with the activities of the other organization. Those activities and their implications for the partnership were flagged for future action, but not integrated into the process. Throughout the development and implementation of the Audacious Partnerships Process, we have tried to grapple with who needs to be involved, in order to, build a transformative partnership. While these are partnerships between organizations, at their core, they are relationships between people. Partnerships need to be comprised of individuals who are both committed to sustainability transformation and influential in their organizations so that partnership lessons can gain purchase outside partnership-specific spaces. We posit that a different kind of partnership is needed between cities and universities. While a normal partnership might facilitate collaboration between the two organizations, what we call a “transformative partnership” would create the foundations necessary for a long-term, sustainability transformation oriented relationship between the two organizations that affects how both organizations function.

The Audacious Partnerships Process targets cities and universities and their potential partnerships as leverage points for urban sustainability transformations (Abson et al. 2017). This implies an underlying thesis: that without intention, and perhaps even with intention, CUPs do not necessarily create transformational change toward sustainability. Keeler and colleagues (2018) outline some of the ways that CUPs build transformational capacity in City administrations. This research began with the university teams in the Capacities network considering how they can create City-university partnerships capable of delivering on all the contributions outlined in that previous work, which brought together the best workings of many different CUPs. The Audacious Partnerships Process was born from the conviction in this group that better CUPs are possible and necessary for transformation. We analyzed the barriers to long-term and transformation-oriented partnership development and the contributions of good partnerships to transformative capacity building. The Audacious Partnerships Process was the result of this analysis and reflection. The process is not a solution, it is a first step toward holding the conversations necessary to build better partnerships. It is a first step toward defining shared values and sustainability goals. It is a first step toward aligning collaborative project development with long-term transformational thinking. More steps need to be taken.

### Limitations

These insights are limited by proximity to the observed events. While there were discussions among university researchers at the four institutions for more than a year after the process was implemented, long-term effects of the process require long-term observation. However, the process itself can be evaluated for its contribution to outcomes that have been shown elsewhere to contribute to lasting change. The process was designed to organize around enabling factors of CUPs through mechanisms previously demonstrated or hypothesized to be foundational to long-term partnership development and sustainability transformation. Additionally, each case intervened in an existing partnership between a City and a university. While we have tried to elaborate how the partnerships functioned before the intervention, we cannot claim that the process resulted in specific sustainability outcomes that would not have otherwise happened without our intervention. We present the Audacious Partnerships Process, a case analysis and partnership survey, to demonstrate how the process can contribute to transformative partnership development in different kinds of CUPs at different stages of partnership development.

## Conclusion

The world is facing an increasing and accelerating set of complex sustainability challenges. To make significant and durable progress on these challenges, urgency must be coupled with long-term strategy. This requires governments, universities, civil society organizations and others to consider how they work together over decades to achieve radical change. The Audacious Partnership Process focused on City-university partnerships – experimenting with how to create new spaces for organizing around long-term sustainability transformations. Going through the process with four City-university partnerships resulted in a series of immediate impacts that indicate a plausible reorienting toward prioritization of long-term partnerships for urban sustainability transformation. Understanding the full impact of this work on City-university partnerships will require further observation and experimentation. However, the process provides a formula for how these organizations might approach one another with the goal of leveraging their shared institutional powers to better address the sustainability challenges faced by their shared communities.

## Supplementary Information

**Additional file 1.** Virtual Game Facilitators Guide.

## Data Availability

All data and materials related to the AudaCITY for Partnerships Process are available upon request or in the supplementary material.

## Abbreviations

APP: Audacious Partnerships Process
ASU: Arizona State University
CUP: City-University Partnership
DCU: Dublin City University
KCL: King’s College London
PSU: Portland State University
LANCIS-IE: National Laboratory of Sustainability Science, Institute of Ecology
UNAM: National Autonomous University of Mexico

## Footnotes

1 The deconstruction ordinance is available at: https://www.portland.gov/bps/climate-action/decon/deconstruction-requirements#:~:text=On%20July%206%2C%202016%2C%20Portland,as%20opposed%20to%20mechanically%20demolished.

## References

[CR1] Abson DJ, Fischer J, Leventon J, Newig J, Schomerus T, Vilsmaier U, Lang DJ (2017). Leverage points for sustainability transformation. Ambio.

[CR2] Allen J, Beaudoin F, Gilden B (2017). Building powerful partnerships: lessons from Portland’s climate action collaborative. Sustainability J Rec.

[CR3] Ansell C, Gash A (2008). Collaborative governance in theory and practice. J Public Adm Res Theory.

[CR4] AtKisson A (2012). The sustainability transformation: how to accelerate positive change in challenging times.

[CR5] Avelino F (2017). Power in sustainability transitions: Analyzing power and (dis) empowerment in transformative change towards sustainability. Environ Policy Gov.

[CR6] Avelino F, Rotmans J (2011). A dynamic conceptualization of power for sustainability research. J Clean Prod.

[CR7] Birch E, Perry DC, Taylor HL (2013). Universities as anchor institutions. J High Educ Outreach Engagem.

[CR8] Caprotti F, Cowley R (2017). Interrogating Urban Experiments. Urban Geogr.

[CR9] Caughman L, Withycombe Keeler L, Beaudoin F (2020). Real-Time Evaluation of City-University Partnerships for Sustainability and Resilience. Sustainability.

[CR10] Cuesta-Claros A, Malekpour S, Raven R, Kestin T. Understanding the roles of universities for sustainable development transformations: A framing analysis of university models. Sustainable Development, Online Advance Version. 2021. 10.1002/sd.2247.

[CR11] Evans J, Karvonen A, Raven R, Evans J, Karvonen A, Raven R (2016). The experimental City: New modes and prospects of urban transformation. The experimental City.

[CR12] Evans J, Vácha T, Kok H, Watson K (2021). How Cities Learn: From Experimentation to Transformation. Urban Planning.

[CR13] Ferrer‐Balas D, Adachi J, Banas S, Davidson CI, Hoshikoshi A, Mishra A, Ostwald M. An international comparative analysis of sustainability transformation across seven universities. Int J Sustain Higher Educ. 2008;9(3).

[CR14] Giunta N, Thomas ML (2013). ‘Lessons from the community partnerships for older adults integrating assessment and evaluation into partnership initiatives’.

[CR15] Joss S (2015). Sustainable Cities: Governing for Urban Innovation.

[CR16] Kalayjian A, Paloutzian RF (2009). Forgiveness and reconciliation.

[CR17] Keeler LW, Beaudoin F, Lerner AM, John B, Beecroft R, Tamm K, Wiek A, Lang DJ (2018). Transferring sustainability solutions across contexts through City-University partnerships. Sustainability.

[CR18] Keeler LW, Beaudoin F, Lerner AM, John B, Beecroft R, Tamm K, Wiek A, Lang DJ (2019). Building actor-centric transformative capacity through City-university partnerships. Ambio.

[CR19] Keeler LW, Bernstein MJ, Nelson JP, Kay BR. AudaCITY: A Capacity-Building Research Method for Urban Sustainability Transformation. Front Sustainable Cities. 2022;92.

[CR20] Kreuter MW, Lezin NA, Young LA (2000). Evaluating community-based collaborative mechanisms: implications for practitioners. Health Promot Pract.

[CR21] Kooiman J, Jentoft S (2009). Meta-governance: values, norms and principles, and the making of hard choices. Public Administration.

[CR22] Meharg S. Catalysing change agents through research for development. PhD Thesis. Australian National University. 2020. Accessed 9/8/2022 from openresearch-repository.anu.edu.au/bitstream/1885/208174/1/Meharg-2020-PhDthesis.pdf

[CR23] Pattberg P, Wilderberg O (2016). Transnational multistakeholder partnerships for sustainable development: Conditions for success. Ambio.

[CR24] Purcell WM, Henriksen H, Spengler JD. Universities as the engine of transformational sustainability toward delivering the sustainable development goals: “Living labs” for sustainability. Int J Sustain Higher Educ. 2019;20(8).

[CR25] Smith PA (2012). The importance of organizational learning for organizational sustainability. The Learning Organization..

[CR26] Soini K, Jurgilevich A, Pietikäinen J, Korhonen-Kurki K (2018). Universities responding to the call for sustainability: A typology of sustainability centres. J Clean Prod.

[CR27] Temper L, McGarry D, Weber L (2019). From academic to political rigour: Insights from the ‘Tarot’ of transgressive research. Ecol Econ.

[CR28] Trencher G, Yarime M, McCormick KB, Doll CN, Kraines SB (2014). Beyond the third mission: Exploring the emerging university function of co-creation for sustainability. Science and Public Policy.

[CR29] UN. New Urban Agenda, United Nations. 2017. Available from: https://www.habitat3.org/the-new-urban-agenda Accessed 13 Dec 2021.

[CR30] Whitehead M, Raco M (2012). The sustainable City: an obituary? On the future form and prospects. Flint, J.

[CR31] Wiek A, Withycombe L, Redman CL (2011). Key competencies in sustainability: a reference framework for academic program development. Sustain Sci.

[CR32] Wooltorton S, Wilkinson A, Horwitz P, Bahn S, Redmond J, Dooley J. Sustainability and action research in universities: towards knowledge for organisational transformation. Int J Sustain Higher Educ. 2015;16(4).

[CR33] Yarime M, Trencher G, Mino T (2012). Establishing sustainability science in higher education institutions: towards an integration of academic development, institutionalization, and stakeholder collaborations. Sustain Sci.

[CR34] Von Wirth T, Fuenfschilling L, Frantzeskaki N, Coenen L (2019). Impacts of urban living labs on sustainability transitions: Mechanisms and strategies for systemic change through experimentation. Eur Plan Stud.

[CR35] Zielke J, Hepburn P, Thompson M, Southern A (2021). Urban Commoning Under Adverse Conditions: Lessons From a Failed Transdisciplinary Project. Front Sustainable Cities..

