# Supplementary material for: Building transformative city-university sustainability partnerships: the Audacious Partnerships Process
Source: Urban Transform. 2023 Jan 20;5(1):1. doi: 10.1186/s42854-022-00045-5 (PMC9851894; doi:10.1186/s42854-022-00045-5)
Supplement: Supplementary file 1 — Additional file 1. Virtual Game Facilitators Guide. [file 42854_2022_45_MOESM1_ESM.pdf]

Introduction
- Game Overview
- Game Purpose
- Team Profiles
- Part One
  - Finding Shared Values and Desired Goals
  - Supporting Structures
  - Conclusion
- Part Two
  - Explorations
  - Impactful Actions
  - Conclusion

Introduction

Creating a sustainable future requires audacious partnerships. What is an audacious partnership? It is a partnership between two or more organizations who commit to work together to overcome their structural and historic limitations in order to contribute to sustainability transformations. We define sustainability transformations as desirable, radical and non-linear societal changes often entailing fundamental changes of system interactions and feedback, which lead to more sustainable system constellations. We know that research, public administration, activism, and entrepreneurship are all essential to building a sustainable and resilient future. But we also know that each, individually, fails to generate the transformation necessary to reach that future. We need audacious partnerships between universities, cities and civil society if the grand challenges of our time, like climate change, are to be comprehensively addressed.

Game Overview

City and university teams will convene to explore their motivations and goals surrounding their partnership and establish a shared vision, identify what supporting structures their partnership requires and will conclude by deciding which actions they should prioritize.

Teams will walk away with a shared understanding of the values driving the partnership, how those values can be realized through concrete projects and actions that can be taken to continue to build and sustain an audacious partnership over time.

Game Purpose

Audacious Partnerships takes city and university partners through the key steps in defining and organizing a partnership that is capable of contributing to sustainability transformations. To

create an audacious partnership, partners must come together to jointly build a foundation that can sustain and adapt for long-term change. They will:

1) Identify the values, both shared and individual, that drive partners to seek a partnership as an important means of addressing shared sustainability challenges (Normative Competence and Interpersonal Competence).
2) Understand the institutional arrangements that influence the behavior of both partners related to the partnerships (Systems Thinking Competence). *Individuals' behavior in partnerships is partly a function of the requirements of their organization and organization type (demands on their time, norms for behavior, existing time horizons for planning and action, expectations for delivery, etc).*
3) Define shared goals for the partnership that are ambitious and push the partnership toward transformation. (Normative Competence)
4) Outline a 10-year vision for the partnership and the role of each organization in the partnership that addresses institutional constraint and achieves the shared goals. (Normative and Futures Thinking Competence)
5) Identify responsibilities, practices and norms for the partnership that reinforce shared values and move the partnership toward the vision over time. (Strategic and Interpersonal Competence)

Overview

The Audacious Partnerships game is part of is a structured process to address the historic and structural limitations of existing organizations through the creation of new and transformational partnerships. The objective is to enable sustainability transformations by creating or re-creating partnerships around audacious partnership goals that are upheld by supporting structures, reinforced and sustained through impactful actions, resilient to future challenges, and owned by individuals and teams through their roles and responsibilities.

This game is an output of CapaCities, an international network of city-university partnerships established in 2016 to accelerate the sustainability transformation of cities. Together, the CapaCities network has created a series of games that build capacity at cities and universities to partner on sustainability projects that lead to change in the communities they serve. The Audacious Partnerships game is the newest CapaCities game, created to support the development of lasting and impactful city-university partnerships. Audacious Partnerships is the result of two years of research and experimentation on partnership development and support for long-term sustainability transformation. We hope you enjoy the game!

Team Profiles

Overview

To begin, each team will share their (4B) *Team Profile* that was completed at the end of the (3B) *Virtual Prework* activity.

The purpose of the Team Profile is to introduce the teams to one another and share the outcomes of the prework. This should help to establish a safe and open environment for game play.

Directions

1. Open your (4B) *Team Profile [INSERT YOUR MURAL LINK HERE].*
2. Using zoom screen share (instead of having each person navigate to the mural link), present the information to your partners.
3. In order to balance participation, have each person introduce themselves.
4. A designated member of each team can share the remaining information regarding team values and goals.

Part One

Finding Shared Values and Desired Goals

Overview

In this round, you will introduce each other to the visions and core values that will animate this partnership. To build your capacity in thinking about sustainability transformations, you will engage in some creative and impromptu storytelling to build your capacity to consider how each other's shared values and desired goals can inform the development of collaborative projects within your partnership. As you build your audacious partnership, it is important for each partner to understand the values and priorities of the other partner and this understanding can be used as a filter for the many opportunities that might seemingly fit within the partnership. In the Audacious Partnerships prework, you were told that "there is a grant opportunity" is not a satisfactory response to the question "What drives you to seek out this partnership?" What drives you to seek at this partnership is more closely tied to the values and Goals you've identified. In this round, you will use these Values and Goals to consider how potential projects could reflect these elements and bring them to life.

The purpose of this round is to establish shared values and desired goals for the partnership and to build your capacity to use these goals and values to consider what projects ought to be pursued and how they ought to function.

Directions

1) Insert the link for the (2C) *Virtual Game* [INSERT YOUR MURAL LINK HERE] into the zoom chat.
2) Ensure that everyone is able to navigate to the board.
3) Select a representative from each team to identify their teams *Values* and *Goals*.
2) Have each representative drag their team *Values* and *Goals* into their indicated space. For shared *Values* and *Goals*, use the "Shared" box instead of duplicating any cards.
3) Read the following directions aloud to the group and have the team representative answer the prompts (these are also displayed on the mural board):
Each team will share their selections and answer the following:
    1) What do you understand each value to mean and why is fundamental to this partnership
    2) What motivated you to select the Goals you selected? How can they serve to orient collaboration within this partnership?

4) After sharing is completed, ask if anyone has any clarifying questions.
5) Next, explain that everyone will practice stretching their thinking about sustainability transformations by completing a storytelling exercise.
6) Read the directions aloud (also displayed on the mural board):

   IT IS 10 YEARS IN THE FUTURE AND YOUR CITY-UNIVERSITY PARTNERSHIP IS FLOURISHING . . .

   1. *Give a brief description of the project and collaboration within your partnership that upholds these 2 Values and 1 Goal.*
   2. *Try to stretch your thinking and be ambitious.*
   3. *Share aloud.*
7) Do your best to randomize, and drag 2 *Values* and 1 *Goal* into the boxes.
8) If you think the group may need help getting started, offer an example of your own.

   Sample: *"10 years ago we began our revision of the climate action plan. Based on the Value #1 Justice, we began by engaging frontline community members from BIPOC communities who are most affected by climate change. Based on Value #2 Trust, we reached out to community-based organizations in order to build relationships to help to overcome a well-earned history of mistrust that BIPOC community members may feel toward large public institutions. This has resulted in Goal #1 Equitable City where our climate resiliency is a tool for racial justice because it is centered on lived experiences and seeks to address the historic trauma of BIPOC community members.*
9) Have each team repeat this exercise until all of the cards have been included in a storytelling round. If time allows, have each team member take a turn.
10) After the storytelling has concluded, ask the group if any *Values* and *Goals* are clear choices (starting with any *Values* and *Goals* that were shared can be a good start).
11) Keeping in mind that you would like to build the groups' capacity for organic dialogue, see if there is group-led momentum to begin the final selections. Feel free to use your facilitative dialogue to move the group toward consensus.
12) If total consensus cannot be reached, call for a vote for the remaining cards.
13) Have players drag the final *Values* and *Goals* to the "PARTNERSHIP GOALS & VALUES" box.

End Result

Teams will have collectively selected 2 Values and 2 Goals that represent a shared foundation for the partnership to build on.

Supporting Structures

Overview

Now that you have selected an initial set of shared values and desired goals to orient your partnership, this round will guide you in prioritizing mechanisms that can support the long-term maintenance of your partnership. These *Supporting Structures* depicted on the cards were developed based on research on city-university partnerships and what is required to sustain and build collaborations that are durable and make progress on sustainability transformations over time. *Supporting Structures* are the pillars of an Audacious Partnership. These structures work

synergistically and therefore they are all essential components for the partnership's growth, adaptation and strength over time.

In this round, you will review the *Supporting Structures* and make short pitches to one another in an effort to determine how your partnership prioritizes these mechanisms. The structures with the highest score will become the *Supporting Structures* that your partnership has identified as priorities.

The purpose of this round is to and to give players the opportunity to talk to one another about why supporting structures are important to them and their organization and to prioritize the supporting structures that can help ensure this partnership is successful in the long term.

Directions

(1) Read all six Supporting Structures Cards aloud to the group to ensure shared understanding and familiarity to each card.
(2) Sort players into 6 groups. Note,
    (a) Breakout rooms may be created in advance in zoom.
    (b) You must use the manual sort function (instead of random) a total of 5 times.
    (c) If you do not create your zoom breakout rooms in advance, at minimum, you should create a list of the player combinations for the five rounds.
    (d) Group size may vary, and there may be a single person in a group.
    (e) If pairing up is required, try to create groups with a balanced distribution of players from the city and the university. If there are less players than cards some players can be responsible for scoring two cards.
(3) Once the groups are identified, assign a single *Supporting Structure* card per group.
(4) You will begin the discussion by telling people they will be placed in breakout rooms.
(5) Once there, each group will read the card aloud to their partner/s while considering the following guiding question (also displayed on the mural board):
    - *How important are each of these supporting structures for enabling your partnership to thrive?*
(6) Each player will work with their partner/s to decide how to distribute 7 points between the two cards. Distribution of the 7 points is based on the relative importance of the two supporting structures.
    (a) Note: half points are not allowed, whereas assigning 0 to one card is allowed.
(7) Once players have agreed on the distribution of points, have them type the number on their corresponding sticky note next to number 1.
(8) Repeat Steps 4-6 until each person/pair has partnered with every other person/pair.
(9) Read the following questions aloud to the group and ask them to keep these in mind as they begin to compare.
(10) The two cards with the highest score will become the priority structures for the partnership.
(11) Have players drag the final *Supporting Structures* to the "PARTNERSHIP SUPPORTING STRUCTURES" box.

Below is an example of what a single completed card will look like after completing 5 rounds with the other cards. This diagram was originally created for the in-person game, but the mechanics remain the same for the virtual version.

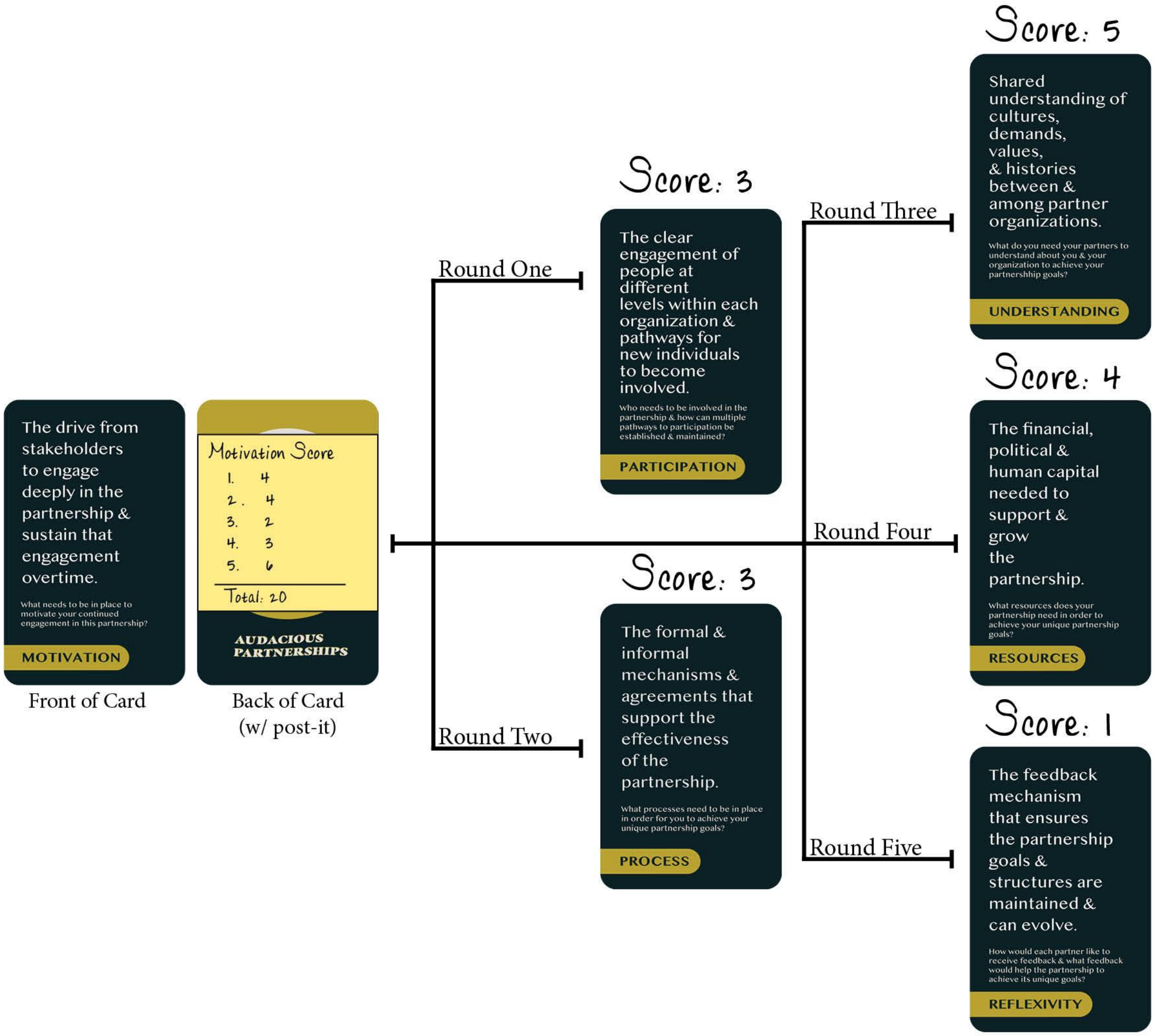

Note, this activity may seem complicated, but the mechanics are quite intuitive Anticipate questions and possibly some trepidation, at first, but once the activity commences, the subsequent rounds should flow easily.

End Result
The partnership will now have identified their unique foundation for their sustainability transformations: 2 Values, 2 Goals, and 2 Supporting Structures, which will serve as the foundation and guidance for their work together.

Conclusion

Have each player complete *Round One Reflections.* This can be done silently and simultaneously.

Part Two

After identifying the *Values, Goals,* and *Supporting Structures* for the partnership, teams will continue to build upon their enhanced understanding in order to brainstorm and propose actions.

Explorations

Overview
In this brainstorming and context exploration activity, players will select prompts that they would like other players to answer. Participants will use the results from *Explorations* to mine potential projects, next steps, and to explore the context surrounding their work. A notetaker will record what is shared in order to aid the next phase of prioritizing actions.

The purpose of this round is for teams to share their experiences in projects. Debrief after the gameplay can help to unpack some of the experiences that were shared in rapid fire.

Directions

1. Share that gameplay will commence over three rounds.
2. Explain that this is a rapid-fire activity, and that players should push themselves to respond quickly.
3. Ask the players to read the 12 *Explorations* to themselves in the #1 grouping.
4. Explain that they will be called upon by another player to fill in a blank on one of the cards. If they provide an answer fast enough, they will call out the next question and select another player.
5. The goal is to try and string as many fast responses together, as possible.
    a. Example: As the facilitator, you start by calling on Jim. Jim has 5 seconds to read a card aloud, and to call on another player to respond (“This partnership’s strength is _________. Beth!”). Beth now has 5 seconds to answer. (“This partnership’s strength is the length of time we have been collaborating!”). Beth now has 5 seconds to call on someone else. If anyone fails to answer fast enough, the facilitator starts back up by calling on a player who has yet to go.
6. Play for grouping #1 ends when each person has had a chance to answer a prompt.
7. Play repeats for groupings #2 and #3
8. Record the responses on sticky notes in the identified box. Be sure to include the response, as well as the player who said it.
9. After all three parts have been cycled through, ask players to record something new that they learned in the “Reflection” box.

End Result
Participants will have listed details and ideas establishing a clearer understanding of their unique and shared contexts. Notetakers will record the details with the intention of aiding in the next steps of selecting Impactful Actions.

Impactful Actions

Overview
Participants will propose Impactful Actions the partnership should pursue. As a group, you will map them and assess how many of your partnership's *Values, Goals and Supporting Structures* are represented, supported or advanced by the Impactful Action.

The purpose of this round is to identify and prioritize actions, with responsible persons, to be taken after game play is complete.

Directions
1. Drag the 6 *Values, Goals* and *Supporting Structures* to the indicated boxes beneath the Audacious Partnerships board.
2. Ask the group "Considering what came up from using the Explorations round, and what you have already been thinking about, **what are some impactful actions you propose the group takes to build the partnership?"**
3. Ask a player to start by selecting an Impactful Action triangle that they would like the group to prioritize. Impactful Actions are sorted by color in 5 categories:
    i. Evaluation and Design
    ii. Engagement
    iii. Training and Capacity Building
    iv. Resources
    v. Communication
4. Tell the player to read the proposed *Impactful Action* aloud, noting to be as specific as possible (e.g. instead of "apply for external" could be elaborated as "apply for a grant from X Funder on climate resiliency).
5. Have them place the *Impactful Action* on the Audacious Partnerships board in the corresponding category.
6. Lead the group in a dialogue regarding which of the 6 *Values* (2), *Goals* (2) and *Supporting Structures* (2) this *Impactful Action* operationalizes or works towards. Through consensus, players will place a star on the board of the corresponding color for the *Values*, *Goals*, and *Structures* it represents or supports.

End Result
Participants will have the opportunity to propose *Impactful Actions* and have the group collectively evaluate them according to the partnerships' *Values, Goals* and *Supporting Structures*. Additionally, they will understand what type of activities they are prioritizing.

Conclusion

Players will conclude gameplay by answering the displayed questions:
1) Decide on a name for your partnership.
2) List an individual and organizational barrier that we must overcome to achieve this transformation.

3) When will you all meet next?
    a) In advance, update the calendar for the month following gameplay.

Then, navigate to the (4C) *Partnership Profile* [INSERT YOUR MURAL LINK HERE]. Using zoom screen share, move the pieces to indicate the players selections, and record their explanations in the corresponding boxes.
